# Incidence of Viral Rebound After Treatment With Nirmatrelvir-Ritonavir and Molnupiravir

**DOI:** 10.1001/jamanetworkopen.2022.45086

**Published:** 2022-12-06

**Authors:** Grace Lai-Hung Wong, Terry Cheuk-Fung Yip, Mandy Sze-Man Lai, Vincent Wai-Sun Wong, David Shu-Cheong Hui, Grace Chung-Yan Lui

**Affiliations:** 1 Department of Medicine and Therapeutics, The Chinese University of Hong Kong, Hong Kong Special Administrative Region, China; 2 Medical Data Analytics Centre, The Chinese University of Hong Kong, Hong Kong Special Administrative Region, China; 3 Institute of Digestive Disease, The Chinese University of Hong Kong, Hong Kong Special Administrative Region, China; 4 Stanley Ho Centre for Emerging Infectious Diseases, Jockey Club School of Public Health & Primary Care, The Chinese University of Hong Kong, Hong Kong Special Administrative Region, China

## Abstract

**Importance:**

Some patients treated with nirmatrelvir-ritonavir have experienced rebound of COVID-19 infections and symptoms; however, data are scarce on whether viral rebound also occurs in patients with COVID-19 receiving or not receiving molnupiravir.

**Objective:**

To examine the incidence of viral rebound in patients with COVID-19 who were treated with the oral antiviral agents nirmatrelvir-ritonavir and molnupiravir.

**Design, Setting, and Participants:**

This cohort study identified 41 255 patients with COVID-19 who were hospitalized from January 1, 2022, to March 31, 2022, in Hong Kong and assessed 12 629 patients with serial cycle threshold (Ct) values measured. Patients were followed up until the occurrence of the clinical end point of interest, death, date of data retrieval (July 31, 2022), or up to 30 days of follow-up, whichever came first.

**Exposures:**

Molnupiravir or nirmatrelvir-ritonavir treatment.

**Main Outcomes and Measures:**

Viral rebound, defined as a Ct value greater than 40 that decreased to 40 or less.

**Results:**

Of 12 629 patients (mean [SD] age, 65.4 [20.9] years; 6624 [52.5%] male), 11 688 (92.5%) were oral antiviral nonusers, 746 (5.9%) were molnupiravir users, and 195 (1.5%) were nirmatrelvir-ritonavir users. Compared with nonusers, oral antiviral users were older, had more comorbidities, and had lower complete vaccination rates. The mean (SD) baseline Ct value was slightly higher in nirmatrelvir-ritonavir users (22.2 [6.0]) than nonusers (21.0 [5.4]) and molnupiravir users (20.9 [5.4]) (*P* = .04). Viral rebound occurred in 68 nonusers (0.6%), 2 nirmatrelvir-ritonavir users (1.0%), and 6 molnupiravir users (0.8%). Among 76 patients with viral rebound, 12 of 68 nonusers, 1 of 6 molnupiravir users, and neither of the nirmatrelvir-ritonavir users died of COVID-19.

**Conclusions and Relevance:**

In this cohort study, viral rebound was uncommon in patients taking molnupiravir or nirmatrelvir-ritonavir and was not associated with increased risk of mortality. Given these findings, novel oral antivirals should be considered as a treatment for more patients with COVID-19 in the early phase of the infection.

## Key Points

**Question:**

What is the incidence of viral rebound after treatment with nirmatrelvir-ritonavir and molnupiravir?

**Findings:**

In this cohort study of 12 629 adults in Hong Kong with COVID-19 who were hospitalized and had serial cycle threshold values measured, viral rebound (defined as a cycle threshold value >40 that decreased to ≤40) occurred in 68 antiviral nonusers (0.6%), 2 (1.0%) nirmatrelvir-ritonavir users, and 6 (0.8%) molnupiravir users.

**Meaning:**

In this study, viral rebound was uncommon in adults with COVID-19 after treatment with nirmatrelvir-ritonavir and molnupiravir, suggesting that these novel oral antivirals should be prescribed to more patients with COVID-19 in the early phase of the infection.

## Teaser

This cohort study of patients with COVID-19 compares the incidence rates of viral rebound in patients with COVID-19 treated with molnupiravir or nirmatrelvir-ritonavir.

## Introduction

Since 2 oral antiviral agents, molnupiravir and nirmatrelvir-ritonavir, became available in late 2021, millions of patients with COVID-19 have received these medications in order to reduce hospitalization rates or adverse clinical outcomes. Evolving data indicate that some patients treated with nirmatrelvir-ritonavir experienced rebound of COVID-19 infections and symptoms after completing the 5-day course.^1^ In May 2022, the US Centers for Disease Control and Prevention (CDC) issued a Health Alert Network Health Advisory to update the public on the potential for COVID-19 rebound after nirmatrelvir-ritonavir treatment.^2^ However, data are scarce on whether viral rebound would also occur in molnupiravir-treated or even untreated patients with COVID-19. In this territory-wide study in Hong Kong, we aimed to examine the incidence rates of viral rebound in patients with COVID-19 receiving molnupiravir, nirmatrelvir-ritonavir, or neither in a community setting and whether viral rebound is associated with clinical outcomes.

## Methods

A territory-wide, retrospective cohort study was performed using the Clinical Data Analysis and Reporting System (CDARS) managed by Hospital Authority, Hong Kong, with which multiple studies on COVID-19 have previously been conducted.^3,4,5,6^ We first identified 41 255 patients with COVID-19 who were hospitalized from January 1, 2022, to March 31, 2022. Of these patients, 12 629 who attended public hospitals with serial cycle threshold (Ct) values measured at least 3 times were included in the analysis (eFigure 1 in Supplement 1).^7^ Patients were followed up until the occurrence of the clinical end point of interest, death, date of data retrieval (July 31, 2022), or up to 30 days of follow-up, whichever came first. The study protocol was approved by the Joint Chinese University of Hong Kong New Territories East Cluster Clinical Research Ethics Committee. A waiver of informed consent was granted by the ethics committee because no patient identifiers were collected. The study followed the Strengthening the Reporting of Observational Studies in Epidemiology (STROBE) reporting guideline.

The primary end point was viral rebound, defined as Ct values greater than 40 that decreased to 40 or lower, as reported in previous studies.^8,9^ The secondary end points were Ct values greater than 36 that decreased to 36 or lower and all-cause mortality.

### Statistical Analysis

Continuous variables are expressed as mean (SD) or median (IQR), as appropriate, and categorical variables are presented as number (percentage). Qualitative and quantitative differences between groups were analyzed by the χ^2^ test or Fisher exact tests for categorical parameters and the *t* test or Mann-Whitney test for continuous parameters, as appropriate. A 2-sided *P* < .05 was considered to be statistically significant. Data were analyzed using R, version 4.1.3 (R Foundation).

## Results

### Patients and Clinical Characteristics

Of the 12 629 patients analyzed (mean [SD] age, 65.4 [20.9] years; 6624 [52.5%] male and 6005 [47.5%] female), 11 688 (92.5%) were oral antiviral nonusers, 746 (5.9%) were molnupiravir users, and 195 (1.5%) were nirmatrelvir-ritonavir users. At baseline, compared with antiviral nonusers, molnupiravir or nirmatrelvir-ritonavir users were older and had more comorbidities, including digestive diseases, diabetes, history of malignant tumor, and lower complete vaccination rate (Table 1). Compared with nirmatrelvir-ritonavir users, molnupiravir users were older and had more cardiovascular diseases, diabetes, cerebrovascular events, respiratory diseases, and kidney diseases, as well as a lower complete vaccination rate (Table 1). The overall case-fatality rate (CFR) was 10.0% (95% CI, 9.5%-10.5%), with CFRs of 13.4% (95% CI, 11.2%-16.1%) for molnupiravir users, 9.7% (95% CI, 6.4%-14.9%) for nirmatrelvir-ritonavir users, and 9.8% (95% CI, 9.3%-10.4%) for nonusers (Table 2).

**Table 1.** Baseline Clinical Characteristics of 12 629 Hospitalized Patients With COVID-19 in the Fifth Wave of COVID-19 Infection in Hong Kong

Clinical characteristic^a^	All (N = 12 629)	COVID-19 oral antiviral nonusers (n = 11 688)	Molnupiravir users (n = 746)	Nirmatrelvir-ritonavir users (n = 195)	*P* value
Age, mean (SD), y	65.4 (20.9)	64.5 (21.0)	78.1 (14.4)	75.7 (14.4)	<.001
Sex					
Male	6624 (52.5)	6105 (52.2)	409 (54.8)	110 (56.4)	.21
Female	6005 (47.5)	5583 (47.8)	337 (45.2)	85 (43.6)	.21
Comorbidities					
Cardiovascular diseases	6800 (53.8)	6112 (52.3)	564 (75.6)	124 (63.6)	<.001
Digestive diseases	2121 (16.8)	1910 (16.3)	167 (22.4)	44 (22.6)	<.001
Diabetes	3923 (31.1)	3511 (30.0)	343 (46.0)	69 (35.4)	<.001
Malignant tumor	855 (6.8)	732 (6.3)	86 (11.5)	37 (19.0)	<.001
Nervous system diseases	1114 (8.8)	1025 (8.8)	74 (9.9)	15 (7.7)	.48
Respiratory diseases^b^	1413 (11.2)	1264 (10.8)	128 (17.2)	21 (10.8)	<.001
Kidney diseases	1460 (11.6)	1298 (11.1)	149 (20.0)	13 (6.7)	<.001
HIV infection	22 (0.2)	17 (0.1)	2 (0.3)	3 (1.5)	.002
Laboratory parameters, mean (SD)					
Hemoglobin, g/dL	12.4 (2.3)	12.5 (2.2)	11.3 (2.3)	11.7 (2.5)	<.001
WBC count, μL	7200 (4400)	7100 (4200)	8000 (4900)	8100 (7900)	<.001
Ct value at baseline					
Mean (SD)	21.2 (5.6)	21.0 (5.4)	20.9 (5.4)	22.2 (6.0)	.04
Median (IQR)	19.9 (16.8-25.6)	19.7 (16.8-24.4)	19.8 (16.9-24.5)	20.7 (17.2-27.6)	.03
Creatinine, median (IQR), mg/dL	0.90 (0.70-1.24)	0.89 (0.70-1.21)	1.11 (0.80-1.86)	0.92 (0.74-1.18)	<.001
ALT, median (IQR), U/L	21.0 (14.2-32.0)	21.0 (14.4-32.0)	19.0 (13.0-28.5)	20.6 (13.4-30.0)	<.001
Albumin, g/dL	3.54 (0.62)	3.55 (0.62)	3.33 (0.60)	3.40 (0.62)	<.001
Total bilirubin, mg/dL	0.58 (0.87)	0.57 (0.84)	0.65 (1.27)	0.63 (0.43)	.02
Steroid for COVID-19	3988 (31.6)	3528 (30.2)	363 (48.7)	97 (49.7)	<.001
Remdesivir	1906 (15.1)	1766 (15.1)	96 (12.9)	44 (22.6)	.003
Age- and sex-specified complete vaccination rate, mean (SD), %	39.4 (20.7)	40.0 (20.8)	31.5 (17.9)	35.6 (18.6)	<.001
Follow-up duration, mean (SD), d	28.5 (4.8)	28.6 (4.7)	27.7 (5.2)	26.8 (5.4)	<.001
Deaths	1264 (10.0)	1145 (9.8)	100 (13.4)	19 (9.7)	.006

Abbreviations: ALT, alanine aminotransferase; Ct, cycle threshold, WBC, white blood cell.

SI conversion factors: To convert hemoglobin to grams per liter, multiply by 10; WBCs to ×10^9^/L, multiply by 0.001; creatinine to micromoles per liter, multiply by 88.4; ALT to microkatals per liter, multiply by 0.0167; albumin to grams per liter, multiply by 10; and total bilirubin to micromoles per liter, multiply by 17.104.

^a^ Data are presented as number (percentage) of patients unless otherwise indicated.

^b^ Respiratory system disease was defined by *International Classification of Diseases, Ninth Revision, Clinical Modification* (*ICD-9-CM*) diagnosis codes for pneumonia other than COVID-19–related pneumonia (*ICD-9-CM* codes 480-487.0) in the previous 3 months, chronic obstructive pulmonary disease and allied conditions (*ICD-9-CM* codes 490-496), pneumoconioses and other lung diseases caused by external agents (*ICD-9-CM* codes 500-508) in the previous 3 months, and other diseases of the respiratory system (*ICD-9-CM* codes 510-519) in the previous 3 months. All comorbidities were represented as binary parameters. Qualitative and quantitative differences between subgroups were analyzed by the χ^2^ test or Fisher exact test for categorical parameters and 1-way analysis of variance or the Kruskal-Wallis test for continuous parameters, as appropriate.

**Table 2.** Deaths in Patients With Viral Rebound According to Different Definitions

Variable	All patients (N = 12 629)	Use of COVID-19 oral antiviral		Use of molnupiravir (n = 746)	Use of nirmatrelvir-ritonavir (n = 195)
		No (n = 11 688)	Yes (n = 941)		
Total No. of deaths	1264	1145	119	100	19
Overall case-fatality rate, % (95% CI)	10.0 (9.5-10.5)	9.8 (9.3-10.4)	12.6 (10.7-15.0)	13.4 (11.2-16.1)	9.7 (6.4-14.9)
Ct >40 then ≤40, No. (%)					
Anytime	76 (0.6)	68 (0.6)	8 (0.9)	6 (0.8)	2 (1.0)
No. (%) of deaths	13 (17.11)	12 (17.65)	1 (12.5)	1 (16.67)	0
Case-fatality rate in patients with viral rebound, % (95% CI)	17.1 (10.4-28.1)	17.6 (10.6-29.4)	12.5 (2.0-78.5)	16.7 (2.8-99.7)	0
Case-fatality rate in patients without viral rebound, % (95% CI)	10.0 (9.4-10.5)	9.8 (9.3-10.3)	12.6 (10.7-15.0)	13.4 (11.1-16.1)	9.8 (6.4-15.1)
Ct >36 then ≤36 anytime, No. (%)					
Anytime	552 (4.4)	509 (4.4)	43 (4.6)	34 (4.6)	9 (4.6)
No. (%) of deaths	54 (9.8)	48 (9.4)	6 (14.0)	5 (14.7)	1 (11.1)
Case-fatality rate, % (95% CI)	9.8 (7.6-12.6)	9.4 (7.2-12.3)	14.0 (6.6-19.3)	14.7 (6.5-33.0)	11.1 (1.8-70.5)

Abbreviation: Ct, cycle threshold.

### Viral Rebound

The median number of polymerase chain reaction assays in all 3 groups was 4 (IQR, 3-5). The mean (SD) baseline Ct value was slightly higher in nirmatrelvir-ritonavir users (22.2 [6.0]) than nonusers (21.0 [5.4]) and molnupiravir users (20.9 [5.4]) (*P* = .04) (eFigure 2 in Supplement 1). Viral rebound, defined as Ct values greater than 40 that decreased to 40 or less, occurred in 6 molnupiravir users (0.8%), 2 nirmatrelvir-ritonavir users (1.0%), and 68 nonusers (0.6%) (*P* = .56). Most of the viral rebounds occurred 2 to 5 days after the completion of antiviral treatment (Figure). With a lower cutoff of Ct values greater than 36 that decreased to 36 or less, such viral fluctuation occurred in 34 molnupiravir users (4.6%), 9 nirmatrelvir-ritonavir users (4.6%), and 509 nonusers (4.4%) (*P* = .95) (Table 2).

**Figure.**
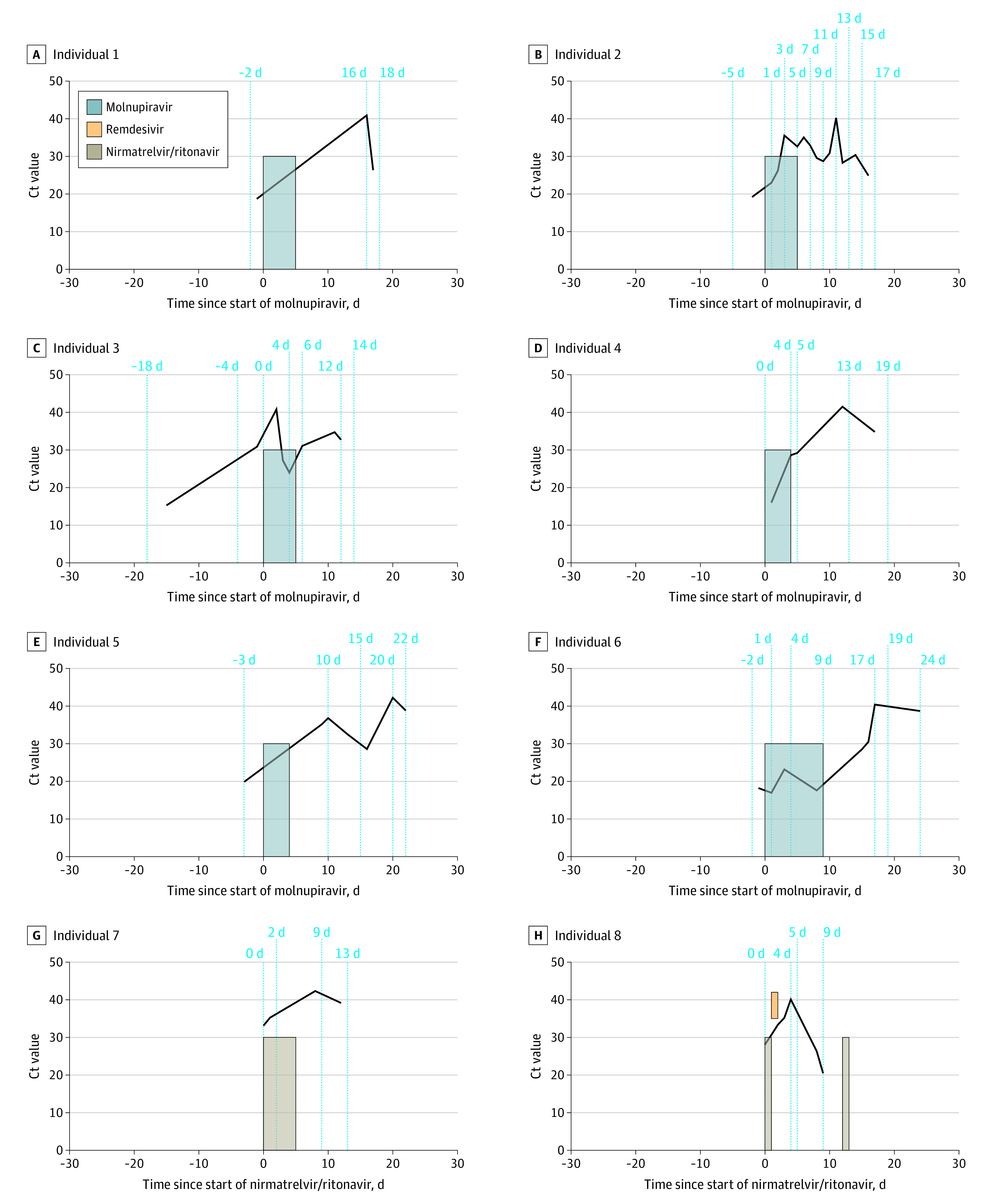
Virologic and Treatment Course of Individuals With Viral Rebound of COVID-19 After Treatment With Molnupiravir or Nirmatrelvir-Ritonavir The width of the bars corresponds to the length of time each patient received treatment. Ct indicates cycle threshold.

### Clinical Outcomes

Among 76 patients with a viral rebound, 12 of the 68 nonusers, 1 of the 6 molnupiravir users, and neither of the nirmatrelvir-ritonavir users died of COVID-19. The CFR among patients with viral rebound was 17.1% (95% CI, 10.4%-28.1%): 16.7% (95% CI, 2.8%-99.7%) for molnupiravir users, 0% for nirmatrelvir-ritonavir users, and 17.6% (95% CI, 10.6%-29.4%) for nonusers (Table 2). Antiviral nonusers with viral rebound had a higher CFR (17.6%; 95% CI, 10.6%-29.4%) than those without viral rebound (9.8%; 95% CI, 9.3%-10.3%), whereas antiviral users with viral rebound (12.5%; 95% CI, 2.0%-78.2%) did not have a higher CFR than those without viral rebound (12.6%; 95% CI, 10.7%-15.0%) (Table 2). Among 552 patients with viral fluctuation, 48 of 509 nonusers and 5 of 34 molnupiravir users died of COVID-19, whereas 1 of the 9 nirmatrelvir-ritonavir users died of pancreatic cancer instead of COVID-19 (Table 2).

## Discussion

To our knowledge, this cohort study is one of the first studies to describe the incidence and clinical outcomes of patients hospitalized for viral rebound of COVID-19 in a community setting. Viral rebound occurred not only in a few nirmatrelvir-ritonavir users but also in molnupiravir users and nonusers. Fortunately, viral rebound did not increase the CFR in molnupiravir users and nirmatrelvir-ritonavir users. The marginal increase in CFR among antiviral nonusers might be associated with the small sample size of patients with viral rebound.

At the critical time of the rapid global spread of the Omicron variant, molnupiravir and nirmatrelvir-ritonavir were approved for outpatient treatment of patients with mild to moderate disease and those at risk for disease progression to reduce the risk of hospital admission and deaths if administered early to high-risk individuals.^10^ In clinical trials, nirmatrelvir-ritonavir demonstrated a greater relative risk reduction in hospitalization and death than molnupiravir compared with placebo.^11,12^ The unique situation in Hong Kong with the availability of both drugs with different antiviral mechanisms at the same time facilitated their contemporary comparisons in a community setting. The early observations of viral rebound with nirmatrelvir-ritonavir^8,9,13^ had raised some concerns in the field. The CDC’s advisory mentioned that such rebound might be part of the natural history of COVID-19 in some persons, independent of antiviral treatment.^2^ At that time (May 2022), limited information was available, and most of the patients who experienced COVID-19 rebound after nirmatrelvir-ritonavir treatment had mild illness without severe disease reported.^14,15^ Hence, the CDC did not recommend additional treatment for COVID-19 for such rebound.

Hong Kong has experienced a fifth wave of COVID-19 in 2022, with a cumulative number of more than 1.4 million cases (ie, nearly 20% of the population) by mid-August 2022. The number of Omicron variant cases increased rapidly from 93% to 100% of all cases since early January 2022. This wave in Hong Kong would be a suitable setting to determine the outcomes of viral rebound in COVID-19 infections caused predominantly by the Omicron variant. In Hong Kong, molnupiravir was preferentially prescribed to more frail patients with multiple comorbidities and polypharmacy than those who received nirmatrelvir-ritonavir, perhaps because of the multiple drug-drug interactions associated with the latter. When nirmatrelvir-ritonavir became available, the guideline had relaxed the use of both oral antivirals in older patients regardless of vaccination status and in younger patients with comorbidities. This guideline change explains why the molnupiravir cohort was older and had more comorbidities than the nirmatrelvir-ritonavir and untreated cohorts. Fortunately, such risk factors of adverse clinical outcomes apparently were not associated with a higher risk of viral rebound, which was equally uncommon in molnupiravir users (0.8%) and nirmatrelvir-ritonavir users (1.0%).

### Strengths and Limitations

The strengths of our study include a territory-wide cohort that covers more than 95% of the inpatient service for patients with COVID-19. All Ct values checked in hospitalized patients were captured by CDARS. The study cohort represents a wider spectrum of patients such that the findings are more representative of individuals encountered in daily clinical practice than those enrolled in clinical trials.

The study also has a few limitations. First, many patients might not be hospitalized at the peak of the fifth wave because of the large numbers of confirmed cases (up to a peak of 70 000 confirmed cases a day), which might lead to fewer hospital admissions as well as fewer Ct values checked. This decreased hospitalization rate might have affected antiviral users and nonusers similarly if they were infected at the same time during the fifth wave. Second, data on symptoms were not available in CDARS, so viral rebound based on laboratory results but not clinical rebound are reported. Third, selection bias may be present because those included in this analysis were patients with serial Ct values monitored and may represent the subgroup with more severe disease or prolonged or repeated hospitalizations. However, the low incidence of viral rebound in this population highlights the rarity of this condition even in severe illness.

## Conclusions

This territory-wide cohort study reports the very low incidences of viral rebound in molnupiravir users, nirmatrelvir-ritonavir users, and antiviral nonusers among patients with COVID-19. Viral rebound is not associated with higher mortality in antiviral users. The study findings support that additional treatment is not necessary for COVID-19 rebound. In view of the ongoing outbreak worldwide, these 2 novel oral antivirals should be prescribed to more patients with COVID-19 in the early phase of the infection, with minimal concerns about viral rebound.

## Notes

Supplement 1. **eFigure 1.** Patient Flowchart **eFigure 2.** Mean Trajectory of Ct Value of Patients With Different Treatment After First Positive Cycle Threshold (Ct) Measurement

Supplement 2.
Data Sharing Statement
