# Supplementary material for: Incidence of Viral Rebound After Treatment With Nirmatrelvir-Ritonavir and Molnupiravir
Source: JAMA Netw Open. 2022 Dec 6;5(12):e2245086. doi: 10.1001/jamanetworkopen.2022.45086 (PMC9856258; doi:10.1001/jamanetworkopen.2022.45086)
Supplement: Supplement 1. — eFigure 1. Patient Flowchart eFigure 2. Mean Trajectory of Ct Value of Patients With Different Treatment After First Positive Cycle Threshold (Ct) Measurement [file jamanetwopen-e2245086-s001.pdf]

Supplementary Online Content

Wong GL-H, Yip TC-F, Lai MS-M, Wong VWS, Hui DSC, Lui GCY. Incidence of viral rebound after treatment with nirmatrelvir-ritonavir and molnupiravir. *JAMA Netw Open*. 2018;1(3):e2245086. doi:10.1001/jamanetworkopen.2022.45086

eFigure 1. Patient Flowchart

eFigure 2. Mean Trajectory of Ct Value of Patients With Different Treatment After First Positive Cycle Threshold (Ct) Measurement

This supplementary material has been provided by the authors to give readers additional information about their work.

© 2022 Wong GL-H et al. *JAMA Network Open.*

eFigure 1. Patient Flowchart

COVID-19 = coronavirus disease 2019, Ct = cycle threshold, SARS-CoV-2 = severe acute respiratory syndrome coronavirus 2.

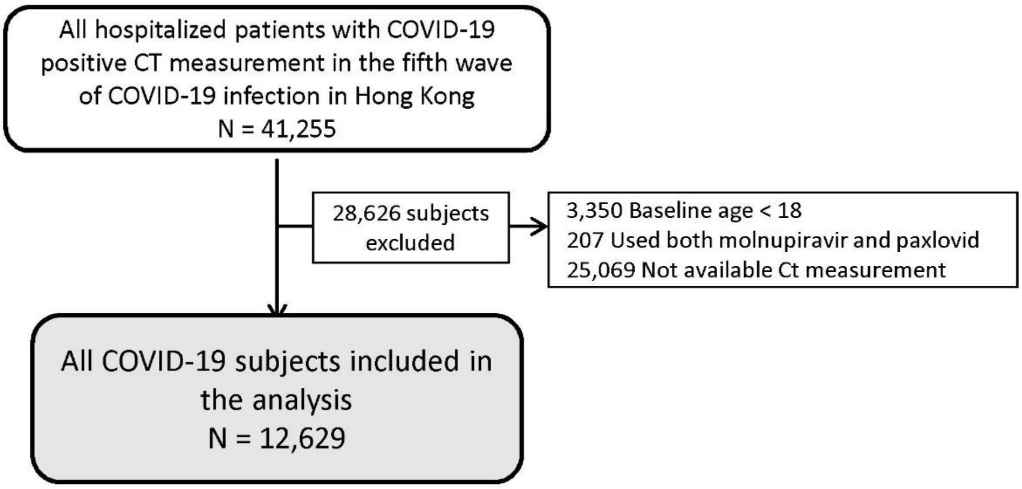

© 2022 Wong GL-H et al. *JAMA Network Open.*

eFigure 2. Mean Trajectory of Ct Value of Patients With Different Treatment After First Positive Cycle Threshold (Ct) Measurement

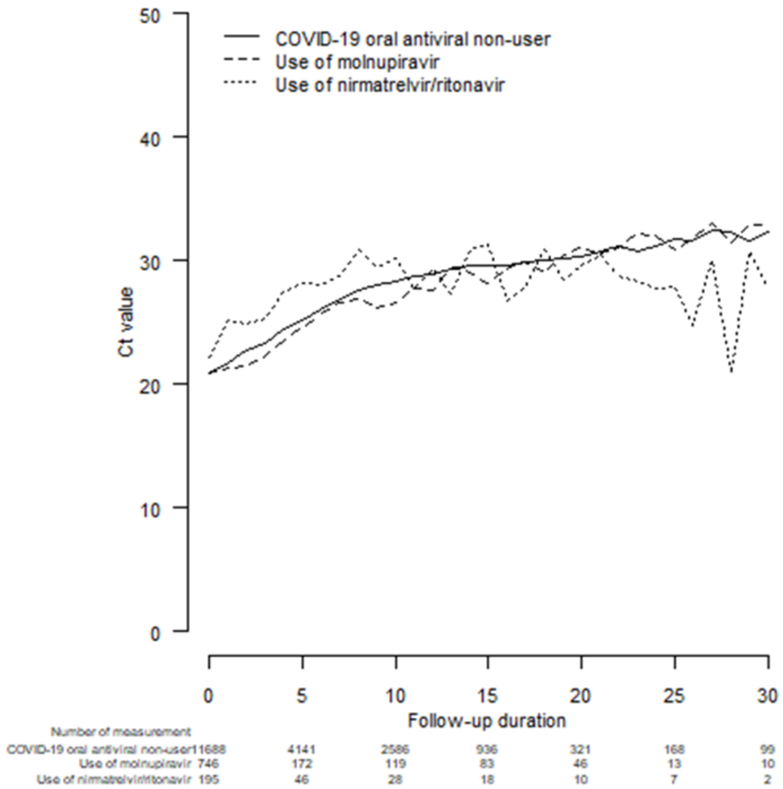

© 2022 Wong GL-H et al. *JAMA Network Open.*
