# Supplementary material for: Incidence of Viral Rebound After Treatment With Nirmatrelvir-Ritonavir and Molnupiravir
Source: JAMA Netw Open. 2022 Dec 6;5(12):e2245086. doi: 10.1001/jamanetworkopen.2022.45086 (PMC9856258; doi:10.1001/jamanetworkopen.2022.45086)
Supplement: Supplement 2. — Data Sharing Statement [file jamanetwopen-e2245086-s002.pdf]

Data Sharing Statement

Wong. Incidence of Viral Rebound After Treatment With Nirmatrelvir-Ritonavir and Molnupiravir. *JAMA Netw Open*. Published December 06, 2022. doi:10.1001/jamanetworkopen.2022.45086

Data
Data available: No

Additional Information
Explanation for why data not available: Data sharing is not allowed due to local regulations.
